# What’s in a Name? Parents’ and Healthcare Professionals’ Preferred Terminology for Pathogenic Variants in Childhood Cancer Predisposition Genes

**DOI:** 10.3390/jpm12081327

**Published:** 2022-08-18

**Authors:** Jacqueline D. Hunter, Eden G. Robertson, Kate Hetherington, David S. Ziegler, Glenn M. Marshall, Judy Kirk, Jonathan M. Marron, Avram E. Denburg, Kristine Barlow-Stewart, Meera Warby, Katherine M. Tucker, Brittany M. Lee, Tracey A. O’Brien, Claire E. Wakefield

**Affiliations:** 1 Discipline of Paediatrics and Child Health, School of Clinical Medicine, UNSW Medicine and Health, UNSW Sydney, Sydney, NSW 2052, Australia; 2 Behavioural Sciences Unit, Kids Cancer Centre, Sydney Children’s Hospital, Randwick, NSW 2031, Australia; 3 Department of Global Pediatric Medicine, St Jude Children’s Research Hospital, Memphis, TN 38105, USA; 4 Children’s Cancer Institute, UNSW Sydney, Kensington, NSW 2750, Australia; 5 Kids Cancer Centre, Sydney Children’s Hospital, Randwick, NSW 2031, Australia; 6 The Westmead Institute for Medical Research, Sydney Medical School, University of Sydney, Sydney, NSW 2052, Australia; 7 Familial Cancer Service, Crown Princess Mary Cancer Centre, Westmead Hospital, Sydney, NSW 2145, Australia; 8 Dana-Farber/Boston Children’s Cancer and Blood Disorders Center, Boston, MA 02215, USA; 9 Center for Bioethics, Harvard Medical School, Boston, MA 02115, USA; 10 Division of Haematology/Oncology, Department of Paediatrics, The Hospital for Sick Children, University of Toronto, Toronto, ON M5G 1X8, Canada; 11 Northern Clinical School, Faculty of Medicine and Health, University of Sydney, Sydney, NSW 2052, Australia; 12 Hereditary Cancer Centre, Prince of Wales Hospital, Randwick, NSW 2031, Australia; 13 Seattle Children’s Hospital and Research Institute, Seattle, WA 98101, USA; 14 Division of Hematology/Oncology, Department of Pediatrics, University of Washington School of Medicine, Seattle, WA 98195, USA

**Keywords:** cancer predisposition, terminology, language, pediatric, communication, genomic sequencing

## Abstract

Current literature/guidelines regarding the most appropriate term to communicate a cancer-related disease-causing germline variant in childhood cancer lack consensus. Guidelines also rarely address preferences of patients/families. We aimed to assess preferences of parents of children with cancer, genetics professionals, and pediatric oncologists towards terminology to describe a disease-causing germline variant in childhood cancer. Using semi-structured interviews we asked participants their most/least preferred terms from; ‘faulty gene,’ ‘altered gene,’ ‘gene change,’ and ‘genetic variant,’ analyzing responses with directed content analysis. Twenty-five parents, 6 genetics professionals, and 29 oncologists participated. An equal number of parents most preferred ‘gene change,’ ‘altered gene,’ or ‘genetic variant’ (n = 8/25). Parents least preferred ‘faulty gene’ (n = 18/25). Half the genetics professionals most preferred ‘faulty gene’ (n = 3/6); however this was least preferred by the remaining genetics professionals (n = 3/6). Many oncologists most preferred ‘genetic variant’ (n = 11/29) and least preferred ‘faulty gene’ (n = 19/29). Participants across all groups perceived ‘faulty gene’ as having negative connotations, potentially placing blame/guilt on parents/children. Health professionals described challenges selecting a term that was scientifically accurate, easily understood and not distressing to families. Lack of consensus highlights the need to be guided by families’ preferred terminology, while providing accurate explanations regarding implications of genetic findings.

## 1. Introduction

Up to 16% of childhood cancer diagnoses are associated with pathogenic/likely pathogenic (P/LP) variants in cancer-predisposition genes [1,2,3]. This knowledge, coupled with rapid advances in next-generation sequencing technology, has led to increased accessibility and use of genomic sequencing in childhood cancer care [4].

Genomic sequencing is increasingly used in childhood cancer care with the expanding role of precision medicine in oncology. Precision medicine provides a more personalized approach to cancer treatment through molecular profiling techniques such as somatic genomic tumor profiling and germline genome/exome sequencing [5]. Somatic genomic tumor profiling allows researchers to identify genetic tumor markers which can help estimate prognosis and likelihood of recurrence and targetable somatic genetic variation in tumor tissue [6]. Germline genome/exome sequencing can identify germline variants in cancer-predisposition genes which increase an individual’s lifetime risk of cancer, known as a cancer predisposition syndrome (CPS) [7]. Identifying children with a CPS may improve patient outcomes by enabling more intensive cancer surveillance and prevention strategies, and potentially facilitating targeted therapeutic approaches [8,9]. Knowledge of a CPS in a child followed by parental trio-testing and consequent predictive testing in at-risk relatives can also inform further familial screening, surveillance, and reproductive decision-making [10].

With potential to improve clinical management, genomic sequencing in childhood cancer is being more broadly implemented in practice [11]. Historically, a patient with a P/LP variant in a gene related to disease, including those associated with a CPS, would be referred to as carrying a ‘mutation’ [12]. ‘Mutation’ was originally a neutral word meaning ‘change,’ or to denote a deviation from a standard sequence, irrespective of phenotypic impact [13,14]. More recently, ‘mutation’ has become associated with radiation damage and disease in scientific literature and science fiction in popular culture, creating increasingly negative perceptions [13,14,15,16]. This has caused incorrect public perception that ‘mutation’ is synonymous with disease-causing [14]. There is now a substantial body of evidence suggesting ‘mutation’ is widely misunderstood and carries strong negative connotations to patients and their families [13,17,18,19,20,21,22]. Labeling a patient with a CPS as carrying a ‘mutation’ may therefore lead to negative psychosocial impacts, including increased anxiety and feelings of guilt [20,21]. Despite a shift in recent years toward retiring ‘mutation,’ [14] and moving away from misleading language in oncology specifically [23], some clinical genetics services continue to use this term in patient communications [24].

To minimize potential distress and/or confusion to patients and families, clinicians and researchers have incorporated alternative terminology to describe genetic variation. These include terms such as ‘alteration/altered gene,’ ‘variation/(genetic) variant,’ ‘genetic/gene change,’ and ‘faulty gene/gene fault,’ among others. However, these new terms bring their own challenges. ‘Alteration’ may not be scientifically accurate as it suggests intentional human modification of the gene [20]. ‘Variant’ could be considered genetic jargon, causing greater worry and confusion than commonly understood words like ‘change’ [17]. ‘Change’ may be too neutral, inaccurately portraying the risk of harboring a germline variant with disease implications [25]. Previous research has explored the naming preferences of adults for a cancer-related pathogenic variant, finding ‘faulty gene’ to be the preferred term among cancer patients, community members and men at high-risk of carrying a BRCA1/2 pathogenic variant [25,26]. Yet in childhood cancer, ‘faulty’ may be inappropriate as it could inadvertently imply blame or be perceived as discriminatory [27].

Multiple guidelines have been created to promote more consistent definitions, description, and laboratory reporting of variation in human medical genetics [28,29,30,31] (Table S1). Each differ slightly in terms of recommended terminology, variant classes, and definitions of pathogenicity for each variant class. These guidelines provide a useful framework for health professionals in interpreting, reporting, and describing P/LP variants. However, there is a lack of consensus regarding how to communicate genetic results to families of children with cancer. For a field that is rapidly evolving, scientifically complex, and sometimes misunderstood, incorporating the preferences of patients/families is necessary for best practice in clinical genetics [32]. To fill this gap, we set out to answer:

- What term do parents with a child with cancer prefer to describe P/LP variants in cancer predisposition genes in children, and why?
- What term do genetics health professionals prefer within the childhood cancer setting, and why?
- What term do pediatric oncologists prefer, and why?

## 2. Materials and Methods

**Figure 1:**
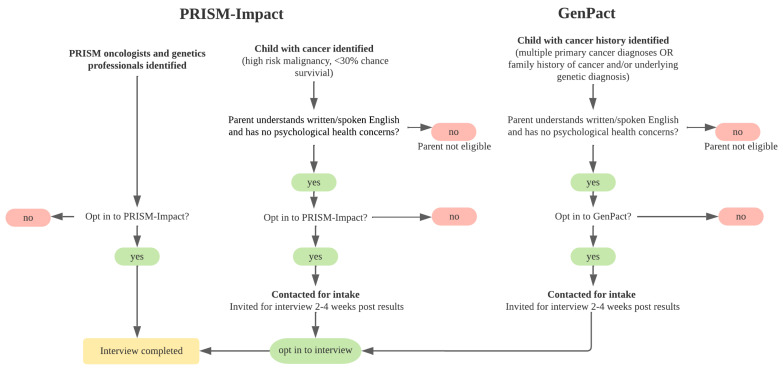
Recruitment schematic.

We collected data from three groups: (i) parents with a child with cancer, (ii) genetics professionals, and (iii) pediatric oncologists. Participants were recruited as part of established research studies: (i) PRISM-Impact, and (ii) GenPact (Figure 1). PRISM-Impact is a prospective, longitudinal psychosocial study which explores experiences of parents with a child with cancer and adolescent patients enrolled in the Australia-wide precision medicine clinical trial for high-risk/poor prognosis malignancies, and healthcare professionals delivering the program [2]. GenPact is a prospective, longitudinal psychosocial study which explores experiences of families whose child has been offered germline genomic sequencing for a current or previous cancer diagnosis, treated at two Australian pediatric hospitals in NSW. The recruitment period for this study spanned June 2020 to May 2022.

We collected qualitative data via semi-structured interviews. We first asked oncologists and genetics professionals to describe what terms/phrasing they use in practice. We then presented all participants with four terms typically used to name P/LP variants in CPS genes: ‘faulty gene,’ ‘altered gene,’ ‘gene change,’ and ‘genetic variant.’ We selected these terms based on a scoping review of literature, previous research by study members [25,26], and consultation with a panel of experts including genetic counselors, clinical geneticists, oncologists, psychologists, and consumers. We asked participants which term they preferred most, least, and their reasons why. We also provided participants an opportunity to suggest any other term they preferred (Table S2).

We collected demographics for health professionals (e.g., years practice) during interviews, and for parents (e.g., age) from their PRISM-Impact or GenPact baseline questionnaire. We extracted patient clinical data (e.g., diagnosis) from study medical databases. Trained psychosocial researchers (JDH., RD, NH) conducted interviews over the phone. We audio-recorded interviews and transcribed verbatim. 

We used the Statistical Package for the Social Sciences (SPSS version 26; IBM, Armonk, NY, USA) to analyze demographic and clinical data via descriptive statistics. We used NVivo (QSR International Pty Ltd., Version 12, 2018, Burlington MA, USA) to conduct an adapted version of a directed qualitative content analysis of interview responses [33]. One psychosocial researcher (JDH) conducted initial coding, applying a coding tree with primary codes for most/least preferred term, and further sub-codes for each suggested term and reasons for preferences. A second psychosocial researcher (RD) conducted a quality check on coded content. There were no discrepancies identified during the quality check to report. 

## 3. Results

### 3.1. Participants

Sixty-six parents, 12 genetics professionals, and 62 pediatric oncologists were invited to participate. Of those, 25/66 parents, 6/12 genetics professionals, and 29/62 pediatric oncologists were interviewed between June 2020 and May 2022. On average, parents were 43.5 years old (SD = 4.9, range = 35–56) and their child was 9.0 years old at diagnosis (SD = 4.9, range = 1–16) (Table 1). On average, genetics professionals had 14.4 years of experience in their field (SD = 6.3, range = 10–25) (Table 2) and pediatric oncologists 19.4 years of experience working in pediatric oncology (SD = 9.5, range = 6–40) (Table 3).

### 3.2. Parents’ Naming Preferences

Twenty-three parents (n = 23/25) nominated at least one term they most and least preferred. Two parents (n = 2/25) endorsed two most preferred terms. Two parents (n = 2/25) had no preference for or against any of the terms, “*I wouldn’t worry about it, none of them bother me*” (mother aged 44, child with sarcoma).

**Figure 2:**
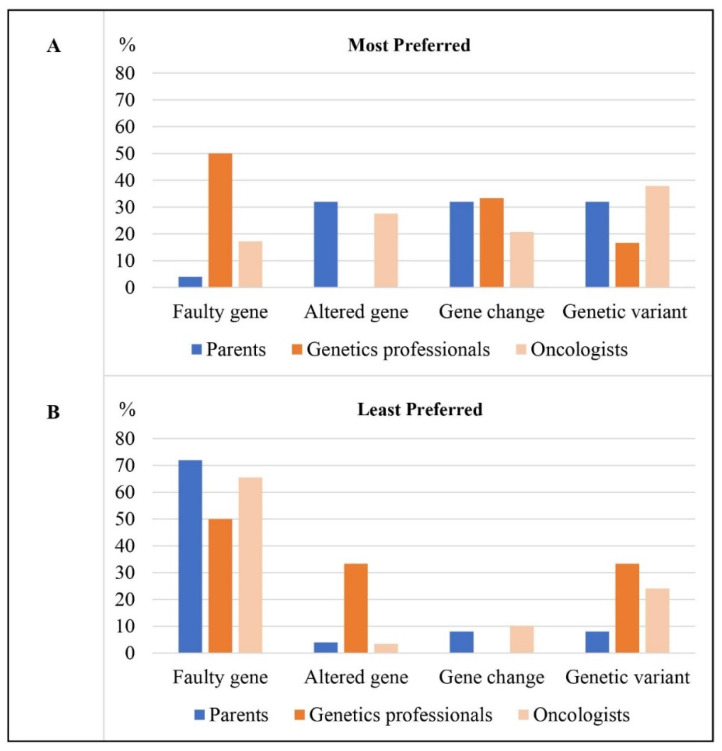
Percentage of participants in each participant group (n = 25 parents, n = 6 genetics professionals, n = 29 oncologists) who (**A**) most preferred each term and (**B**) least preferred each term.

An equal number of parents most preferred the terms ‘gene change,’ ‘genetic variant,’ or ‘altered gene’ (n = 8/25) (Figure 2/Table 4). Parents preferred ‘gene change’ because of the emotional neutrality of the term and/or because it was easy to understand.

“*I think that’s probably the easiest to understand. Plus, probably not as traumatic sounding.*”

(mother aged 42, child with central nervous system tumor)

Parents who most preferred ‘genetic variant’ or ‘altered gene’ described them as less emotive than others.

“*(Genetic variant) sounds like it’s OK like it’s not …as harsh.*”

(mother aged 45, child with central nervous system tumor)

One parent most preferred ‘faulty gene’ (n = 1/25).

For their least preferred term, most parents chose ‘faulty gene’ (n = 18/25) (Figure 2/Table 4). Many shared that they had an immediate negative reaction to ‘faulty gene;’ “*faulty never sounds good*” (mother aged 47, child with central nervous system tumor), “*I had a reaction to the first one—faulty*” (Father aged 47, child with sarcoma). Parents described disliking this term because of its negative connotations, such as implying something negative about their child: “*As a parent you don’t like to… even though you know it’s faulty you don’t like to hear that term*” (mother aged 48, child with thyroid cancer), or implying fault of the parent or child.

“*Not faulty. I must have spent one whole month not sleeping properly thinking how and why [my child got cancer]… so fault would probably just add another pile of extra stuff to the plate.*”

(father aged 49, child with sarcoma)

“*They were very explicit when talking to [child’s name] that…you haven’t done anything bad. They never wanted him to think he had done anything wrong to cause this happening…that sat with me… I don’t like the use of a word that would imply that the person themselves caused that problem to occur.*”

(mother aged 44, child with retinoblastoma)

Other parents rated ‘genetic variant’ (n = 2/25), ‘gene change’ (n = 2/25) or ‘altered gene’ (n = 1/25) as their least preferred term. ‘Genetic variant’ was perceived as too formal or scientific by these parents, whereas ‘gene change’ was described as unfamiliar/unclear. Parents acknowledged that understanding of terms is dependent on each parents’ background, including their cultural/linguistic background, education, and health literacy.

“*[Genetic variant]… I know what it means, but I’ve also come across lots of families in wards that are ESL [English as a second language], such as my husband…those terms are too scientific for him to really grasp what you mean*.”

(mother aged 35, child with Wilms tumor)

When parents were asked if there was another word they prefer, most shared that there was not (n = 21/25). Three parents suggested ‘mutation,’ with one parent describing ‘mutation’ as “*reasonably easy to understand*” (mother aged 43, child with leukemia/lymphoma) and another acknowledging that it was familiar but “*probably not a good one*” (father aged 47, child with sarcoma). One parent described using the term “*wonky cells*” (mother aged 37, child with sarcoma).

### 3.3. Genetics Professionals’ Naming Preferences

All genetics professionals nominated one term they most preferred. Five genetics professionals (n = 5/6) nominated at least one term they least preferred, with one (n = 1/6) least preferring none of the terms provided, choosing ‘mutation,’ as their least preferred term. Two genetics professionals (n = 2/6) endorsed two least preferred terms.

Half the genetics professionals (n = 3/6) most preferred ‘faulty gene’ (Figure 2/Table 4). They highlighted that this term was most logical as it implied that what was found was disease-causing.

“*If you want to make it very clear that it’s a pathogenic variant, a disease-causing variant rather than a change, a fault indicates that it’s incorrect*.”

(genetic counselor, years practice unknown)

This was consistent with what genetics professionals shared they typically use in practice, with two genetics professionals (n = 2/6) spontaneously offering ‘fault/faulty gene’ as the language they would use in clinic. Genetics professionals explained that their choice of terminology would also depend on a family’s specific circumstance. This included factors such as any prior awareness of the finding or language the family were already familiar with.

“*You just have to take it on an individual basis because by the time he got to us he knew there was a gene fault in the family*.”

(clinical geneticist, 25 years practice)

“*I will tend to adapt my language with what I hear the patients saying back to me*.”

(genetic counselor, 12 years practice)

Other genetics professionals most preferred ‘gene change’ (n = 2/6), or ‘genetic variant’ (n = 1/6).

For their least preferred term, half of the genetics professionals chose ‘faulty gene’ (n = 3/6) (Figure 2/Table 4). They described this term as having negative connotations or implying something negative about the child. As one participant who worked with bereaved parents put it, “*It’s their child that you’re talking about, it’s a young child who they loved dearly… it could be seen as you’re calling their child faulty and from that respect it probably has more negative connotations… and they’ve already been through so much*” (genetic counselor, 15 years practice).

An equal number of genetics professionals chose ‘altered gene’ (n = 2/6) and ‘genetic variant’ (n = 2/6) as their least preferred terms. Genetics professionals perceived these terms as difficult to understand, with ‘altered gene’ described as sounding like the gene had been intentionally modified, and ‘genetic variant’ described as too complex, “*variant is just a complex word, for most people it doesn’t make any sense*” (genetic counselor, 10 years practice).

Genetics professionals were asked if there was any other term they may prefer or use. Many shared that there was not (n = 3/6), while others mentioned the term ‘glitch’ (n = 1/6) and the concept of ‘working/non-working gene copies’ (n = 2/6).

### 3.4. Pediatric Oncologists’ Naming Preferences

All oncologists nominated at least one term they most and least preferred. One oncologist (n = 1/29) endorsed two terms they most preferred and one (n = 1/29) endorsed two terms they least preferred.

Many oncologists most preferred ‘genetic variant’ (n = 11/29), perceiving this term as more neutral and less emotive than others: “*it’s gentler*” (pediatric oncologist, 33 years practice) (Figure 2/Table 4). Oncologists also described this term as scientifically accurate and versatile as it could be used in discussions about pathogenic, benign and variants of uncertain significance.

“*I think the most broadly encompassing term would be a genetic variant, one because it describes change in itself, and not necessarily what its implications might be.*”

(pediatric oncologist, 9 years practice)

Other oncologists most preferred ‘altered gene’ (n = 8/29), ‘gene change’ (n = 6/29) or ‘faulty gene’ (n = 5/29). ‘Altered gene’ was described as less technical for parents, without implying fault or negative connotations.

“*I use the word altered. Because I don’t see it as faulty. It’s faulty from the point of view of what it may cause, but it may protect you against something else which I don’t understand*.”

(pediatric oncologist, 10 years practice)

‘Gene change’ was preferred due to it being a broad and neutral term that depicts the gene as different, but “*doesn’t necessarily put a positive or negative spin on it*” (pediatric oncologist, 14 years practice). ‘Faulty gene’ was preferred as it was perceived as easy for parents to understand whilst making it clear that what was found was disease-causing, “*it tells them that it’s not functioning normally and it makes it very clear that it has significance, not only for the child, but for any future offspring*” (pediatric oncologist, 33 years practice). One oncologist mentioned using ‘faulty gene’ because that’s how they had been trained.

When oncologists were asked what terms they typically use in practice, only a few indicated that they would use terms like ‘faulty gene’ (n = 2/29) or ‘variant’ (n = 3/29). Several oncologists mentioned that they typically phrased a disease-related variant in a cancer gene as a ‘mutation’ or ‘abnormality’ (n = 7/29). Oncologists also described how their discussion of germline findings would often require the support of the clinical genetics team, “*explaining… germline findings would be something that would require probably further consultation with our genetic colleagues, especially if there are any potential implications in terms of medical conditions*” (pediatric oncologist, 9 years practice). They described how their language would also depend on families’ level of understanding and specific circumstances, “*sometimes it’s easier, sometimes it’s difficult, depending on the family’s expectations, and depending on how well or unwell the child is doing*” (pediatric oncologist, 15 years practice).

For their least preferred term, most oncologists chose ‘faulty gene’ (n = 19/29) (Figure 2/Table 4). Oncologists shared that they disliked this term because they felt it had inherent negative connotations, implied something negative about the child, and/or placed guilt on parents/child.

“*It sounds very negative…without proper explanation and genetic counseling…it could cause a lot of anxiety, until they are explained what it actually means*.”

(pediatric oncologist, 14 years practice)

“*Telling somebody to their face that you’ve got a faulty gene puts a whole heap of guilt on them…one of the things that comes up particularly when you start talking about hereditary cancer is that I think a lot of parents will take on the guilt that I did this. It’s my fault.*”

(pediatric oncologist, 21 years practice)

‘Genetic variant’ (n = 7/29) was chosen as least preferred by some oncologists who perceived it as too technical and scientific for families to understand, particularly culturally diverse families: “*Genetic variation doesn’t mean anything to a lay person.*” (pediatric oncologist, 30 years practice), “*It could be misunderstood in a pejorative way. We’ve got lots of people in our clinic from a non-English speaking background and you know, language matters*” (pediatric oncologist, 36 years practice). Few oncologists chose ‘gene change’ (n = 3/29) or ‘altered gene’ (n = 1/29) as their least preferred term. Reasons for least preferring these terms included that they were inaccurate, with one oncologist describing ‘gene change’ as “*inaccurate and it’s harsh*” (pediatric oncologist, 33 years practice), and another that it did not “*convey the seriousness of it*” (pediatric oncologist, 33 years practice). They were also both described as incorrectly implying that the gene had been intentionally modified or changed “*the other two suggest that it might have been otherwise and then it changed*” (pediatric oncologist, 33 years practice).

Oncologists were asked whether there was another term that they may prefer or use. Most shared that there was not (n = 15/29). Some reported that they would use ‘mutation’ (n = 4/29), stating that parents often understand it. A broad range of other terms and phrases were also suggested, such as ‘mistakes in the gene,’ ‘abnormal gene,’ and ‘inherited changes’.

## 4. Discussion

We conducted a qualitative study with parents of a child with cancer, genetics professionals, and pediatric oncologists to determine the most appropriate term to communicate with families about P/LP variants in cancer-related genes in children. We found no clear consensus within or among groups for the most preferred term. However, parents more commonly preferred terms they perceived as neutral. Regarding least preferred terms, there was some agreement among groups with most parents, pediatric oncologists, and half of genetics professionals least preferring ‘faulty gene.’ Parents’ preferences were related to their emotional response and ability to understand each term. Health professionals described being conscious of emotional impacts for parents/patients, being scientifically accurate in discussions, and ensuring comprehensive understanding of genetic findings.

Two studies have previously explored naming preferences of patients at increased risk of carrying a cancer-related P/LP germline variant [25,26]. In contrast to our findings, both studies found ‘faulty gene’ received the highest preference rating among patients with cancer. Given both studies were conducted more than 10 years ago, it is conceivable that preferences in the community have changed over time. Both studies also examined terminology preferences in an adult cancer context, potentially highlighting the unique complexities associated with the childhood cancer setting. Parents of children with cancer already experience high levels of blame and guilt associated with their child’s diagnosis [34]. These feelings may be further intensified by discussions around genetic testing and transmission of disease-risk to children [35,36]. Another previous study asked attendees at a genetics lay-advocacy conference, which included adults with genetic conditions, to give their preferences and opinions on controversial genetic terms [21]. This study concluded that language that invokes parental blame/guilt or makes people feel negatively about themselves or their children should be avoided. Our findings support this study, emphasizing the need for neutral terminology, regardless of the term, in the childhood cancer setting.

Genetics professionals and oncologists in this study shared similar concerns as parents regarding ‘faulty gene.’ However, there were notable differences in parent and healthcare professional reasons for least preferring this term. Parents in this study were primarily focused on culpability and blame, whereas health professionals mostly discussed potential negative connotations. This may be due to differences between parent and healthcare professional understanding and perceptions of the term ‘faulty gene.’ Unlike parents, genetics professionals and oncologists recognized faulty gene’ as the only term that accurately implied pathogenicity. Genetics professionals have previously described the challenge of balancing sensitivity and technical accuracy when discussing genetic information with patients [21]. This could explain the lack of consensus among genetics professionals in our study where half least preferred ‘faulty gene’ because it harbored negative connotations, while the other half preferred it because they perceived it as most accurate. One term that could potentially strike this balance is the concept of ‘working/non-working gene copies,’ suggested by genetics professionals in our study. This concept may accurately describe the implications of a P/LP variant, without having negative connotations or emotional impacts. Further research on the acceptability of ‘working/non-working genes’ is needed.

Many oncologists most preferred the term ‘genetic variant,’ perceiving it as neutral, yet accurate. These findings may be indicative of participants’ level of comfort with communicating about genetics. Non-genetics clinicians have previously detailed challenges and a lack of confidence in communicating genomic results to families [37,38,39]. ‘Genetic variant’ is widespread throughout the literature and laboratory reporting [28,29,30,31] and may be the term with which non-genetics clinicians are most familiar. Oncologists in this study indicated challenges communicating about genetics in describing how discussion of germline results with families may require prior consultation with genetics colleagues. While this is a valuable multidisciplinary approach, this needs to be appropriately balanced so as not to place an additional burden on already strained genetics services [40,41]. With increased uptake of genomics into practice as precision medicine becomes more accessible for patients with childhood cancer, education and training for clinicians to improve their confidence discussing genetic findings with families will be important to reduce the reliance on over-burdened genetics services.

It is worth acknowledging the pace at which genomics has become part of household conversation through the COVID-19 pandemic. Widespread use of ‘variant’ in media and public discourse may lead to the term ‘genetic variant’ becoming more familiar to and acceptable among the public over time. Ongoing research is needed in this field to remain current to families’ preferences.

Strengths of our study include providing valuable qualitative insight of multiple stakeholders terminology preferences for P/LP variants in the childhood cancer setting, particularly parents with a child with cancer. However, due to the small sample and qualitative nature of the data, results may not be readily generalizable outside the study population. This small sample size also limited our ability to examine participant factors that may have impacted preferences, such as health professionals years of experience. There was also a low participation rate among all participant groups. In addition to this, our sample was not representative of the wider childhood cancer community in Australia, with parents mostly highly educated, English-speaking, from Western/European cultural backgrounds, and female. Additionally, children in this study mostly had high-risk cancers, and therefore parents may have been sensitive to language due to the severity of their child’s situation. The high-risk nature of their child’s cancer may also have caused parents to be little focused on the implications of carrying a CPS in comparison to their child’s short-term survival. We also acknowledge the possibility that our findings may have been impacted by order-effect bias and response-order bias as a result of the method of questioning used in the interview guide.

This study highlights the importance of considering families’ preferences and level of understanding when discussing genetic results in childhood cancer. Neutral terms may be preferable in the childhood cancer setting to minimize feelings of blame or guilt. ‘Faulty gene’ may have attracted negative connotations for parents of children with cancer and may no longer be appropriate. However, balancing the emotional impact with scientific accuracy and understanding can be challenging for health professionals delivering genetic findings to families. Future research should explore alternative terminology that is free from emotional attachments, such as ‘working/non-working genes.’ Further research assessing preferences and parent understanding in a larger and more representative sample, including greater representation from fathers of children with cancer, would also allow for the identification of factors that may influence participant preferences, and greater consensus on the most appropriate terminology.

## Figures and Tables

**Table 1:** Demographics, parents (n = 25) and patients of participating parents (n = 25).

Characteristic	Data
**Parents**	
Age, mean (SD), range	43.5 (4.9), 35–56
Sex, no. (%)	
Female	22 (88.0)
Education, no. (%)	
Year 12 or below	2 (8.0)
Certificate/Diploma	8 (32.0)
Higher Education	15 (60.0)
Previous genetics education, no. (%)	
Yes	9 (36.0)
No	15 (60.0)
Unsure	1 (4.0)
Self-reported perceived genetic knowledge, no. (%)	
Below average	8 (32.0)
About average	12 (48.0)
Better than average	5 (20.0)
Religion type, no. (%)	
Christian	13 (52.0)
Other religion	4 (16.0)
No religion	8 (32.0)
Cultural background, no. (%)	
Western/European	18 (72.0)
Other	6 (24.0)
Missing	1 (4.0)
Research study child enrolled in, no. (%)	
PRISM	21 (84.0)
GenSeq	4 (16.0)
**Patients**	
Age at study enrolment, mean (SD), range	10.4 (4.8), 1–17
Age at diagnosis, mean (SD), range	9.0 (4.9), 1–16
Time (years) since diagnosis and enrolment, mean (SD), range	1.4 (2.6), 0–10
Sex, no. (%)	
Female	11 (44.0)
Cancer type, no. (%)	
Central nervous system	6 (24.0)
Sarcoma	9 (36.0)
Leukemia/Lymphoma	2 (8.0)
Thyroid	2 (98.0)
Other	6 (24.0)
Reportable germline finding identified via research testing, no (%)	
No	22 (88.0)
Yes	3 (12.0)

SD = Standard Deviation, no. = number, PRISM = PRecIsion Medicine for children with cancer, GenPact = The psychosocial imPACT of GENetic testing in cancer.

**Table 2:** Demographics, genetics professionals (n = 6).

Characteristic	Data
Profession, no. (%)	
Genetic counselor	5 (83.3)
Clinical geneticist	1 (16.7)
Sex, no. (%)	
Female	6 (100)
Age, mean (SD), range (n = 5)	45.8 (9.9), 38–63
Years of practice in genetics, mean (SD), range (n = 5)	14.4 (6.3), 10–25
Percentage time dedicated to research, mean (SD), range (n = 5)	14% (22.7), 2–60
Self-reported formal genetics training, no. (%)	
As part of compulsory genetics training only	5 (83.3)
Missing	1 (16.7)

SD = Standard Deviation, no. = number.

**Table 3:** Demographics, oncologists (n = 29).

Characteristic	Data
Profession, no. (%)	
Pediatric oncologist	29 (100)
Sex, no. (%)	
Female	12 (41.4)
Age, mean (SD), range	50.7 (10.5), 25–76
Years of practice in pediatric oncology, mean (SD), range	19.4 (9.5), 6–40
Percentage time dedicated to research, mean (SD), range	33.6 (20.6), 5–80
Self-reported formal genetics training, no. (%)	
Very little/none	11 (37.9)
As part of compulsory medical training only	11 (37.9)
Additional training (e.g., PhD in genetics)	7 (24.1)

SD = Standard Deviation, no. = number.

**Table 4:** Proportion of participants in each participant group who most and least preferred each term ad key reasons provided.

Term	Preference	Participant Group **			Pros	Cons
		Parents	Genetics Professionals	Oncologists		
Faulty	Most prefer	1/25	3/6	5/29	- Implies what was found is disease causing ^b,c^ - Easily understood by parents ^c^	- Negative connotations to child ^a,b,c^ - May place blame/guilt on parents or child ^a,b,c^
	Least prefer	18/25	3/6	19/29		
Altered gene	Most prefer	8/25	0/6	8/29	- Neutral/less emotive ^a,c^ - Easily understood/not technical ^c^	- Difficult to understand ^b^ - Implies modified gene ^b,c^ - Scientifically inaccurate ^c^
	Least prefer	1/25	2/6	1/29		
Gene change	Most prefer	8/25	2/6	6/29	- Neutral/less emotive ^a,c^ - Easily understood by parents ^a^	- Scientifically inaccurate ^c^ - Implies modified gene ^c^
	Least prefer	2/25	0/6	3/29		
Genetic variant	Most prefer	8/25	1/6	11/29	- Neutral/less emotive ^a,c^ - Scientifically accurate ^c^	- Difficult to understand/too technical ^a,b,c^
	Least prefer	2/25	2/6	7/29		
None of the above	Most prefer	2/25	0/6	0/21	N/A	N/A
	Least prefer	2/25	1/6 *	0/29		

^a^ based on feedback from parents; ^b^ based on feedback from genetics professionals; ^c^ based on feedback from oncologists. * This participant least preferred ‘mutation’ more than any of the four terms provided. ** Participants could suggest more than one term as their most or least preferred term, or indicate that they had no preference, therefore the summation of preferences does not equal the total number of participants.

## Data Availability

The data presented in this study are available on request from the corresponding author. The data are not publicly available due to privacy/ethical reasons.

## Supplementary Materials

The following supporting information can be downloaded at: https://www.mdpi.com/article/10.3390/jpm12081327/s1, Table S1: Summary of existing guidelines for the description of sequence variation in medical genetics; Table S2: Semi-structured interview guide.
