# Supplementary material for: What’s in a Name? Parents’ and Healthcare Professionals’ Preferred Terminology for Pathogenic Variants in Childhood Cancer Predisposition Genes
Source: J Pers Med. 2022 Aug 18;12(8):1327. doi: 10.3390/jpm12081327 (PMC9410181; doi:10.3390/jpm12081327)
Supplement: Supplementary file 1 [file jpm-12-01327-s001.zip › jpm-1828116-supplementary.pdf]

Supplementary Table S1. Summary of existing guidelines for the description of sequence variation in medical genetics.

Author	Original date of publication	Date of revisions	Condition/context	Recommendations	Additional comments
American College of Medical Genetics (ACMG)/Association for Molecular Pathology (AMP)	2000	2008, 2015	Laboratory guidelines for reporting of sequence variants in medical genetics. Originally created as educational resource for clinical laboratory geneticists to help them provide quality clinical laboratory services.	Modifier terms for 'variant' to describe sequence changes with varying levels of evidence of pathogenicity, **OR** evidence of benign.<br><br>**Class** / **Modifier** / **Likelihood of Pathogenicity**<br>1 / Benign / <0.1%<br>2 / Likely benign / 0.1-9.9%<br>3 / Uncertain significance / 10-89.9%<br>4 / Likely pathogenic / 90-99%<br>5 / Pathogenic / >99%	Suggest that 'mutation' and 'polymorphism' be replaced by 'variant.'
Human Genome Variant Society (HGVS)	2000	2016	Internationally accepted standard for the consistent and unambiguous description of sequence variants in molecular	Recommendations relate to naming of mutations at nucleotide, DNA, RNA and protein level descriptions (e.g. location within the gene, type of variation).	Suggest 'polymorphism' and 'mutation' should no longer used be used. State that only neutral terms

<table>
<tr><td></td><td></td><td></td><td>diagnostics. Guidelines relate to naming of variation at nucleotide, DNA, RNA and protein level descriptions.</td><td></td><td>such as ‘variant’ ‘alteration’ and ‘change’ should be used instead of these terms.</td></tr>
<tr><td>International Agency for Research on Cancer (IARC)</td><td>2008</td><td>N/A</td><td>Standardized classification system for application to sequence based results for cancer predisposition genes.</td><td>Modifier terms for ‘variant’ to describe sequence changes of varying pathogenicity:
<table>
<tr><th>Class</th><th>Modifier</th><th>Likelihood of Pathogenicity</th></tr>
<tr><td>1</td><td>Not pathogenic</td><td><0.1%</td></tr>
<tr><td>2</td><td>Likely not pathogenic</td><td>0.1-4.9%</td></tr>
<tr><td>3</td><td>Uncertain</td><td>5-94.9%</td></tr>
<tr><td>4</td><td>Likely pathogenic</td><td>95-99%</td></tr>
<tr><td>5</td><td>Definitely pathogenic</td><td>>99%</td></tr>
</table></td><td></td></tr>
<tr><td>Evidence-Based Network for the Interpretation of Germline Mutant Alleles (ENIGMA)</td><td>2019</td><td>N/A</td><td>Review of vocabulary used for the interpretation and reporting of</td><td>Developed more detailed description of the clinical implications of, and management recommendations associated with germline variants placed in each of the classification tiers</td><td>Highlighted inconsistencies associated with use of the term ‘mutation.’</td></tr>
</table>

			germline genetic tests for cancer susceptibility.	previously described by IARC and ACMG.	

Supplementary Table S2. Semi-structured interview guide

Topic	Questions and prompts	Participant group
Introduction	"Sometimes telling your story may bring up a range of feelings or memories. So, just before we get started I wanted to check in to see how you are feeling. On a scale of 0-10 where 0 is "None" and 10 is "Extreme" how much DISTRESS have you experienced this past week, including today? (followed by anxiety, depression, anger and need for help) [if >8, see risk management protocol at the end of the interview schedule]"<br><br>"Do you have any questions? Please let me know if you are feeling distressed or would like to stop at any point."<br><br>"Just to get to know you a little, could you tell me briefly a little about your child and their experience with cancer?'	parents
Demographics	"Just to start with would you mind sharing some personal demographics with me?"<br>- Year of birth<br>- profession (we should know before interview - just to confirm)<br>- number of years of practice.<br>- what percent of your time is dedicated towards research?<br>- How much, and what kind of formal genetics training have you had, if any? (probe: was it an elective or a compulsory part of your training?)	genetics professionals, oncologists
Phrasing of germline results	"If you directly discussed germline findings with a family, do you recall how you phrased the germline results to the family?"	genetics professionals, oncologists
Genetic terminology preferences	"We would like to know your preference on the language used to describe these types of genes to families. I am going to suggest four terms for	genetics Professionals, oncologists

	this: Faulty gene, altered gene, gene change, or genetic variant?" *[ensure you say these really slowly]*<br>- Of the four terms, which one do you prefer most? (why?)<br>- Which one do you prefer least? (why?)<br>- is there another word you would prefer?	
	"Sometimes changes happen in our genes which cause them to not work properly. This can increase a person's chance of developing cancer. We would like to know your preference on the language we use to describe these types of genes. I am going to suggest four terms for this: Faulty gene, altered gene, gene change, or genetic variant?" *[ensure you say these really slowly]*<br>- Of the four terms, which one do you prefer most? (why?)<br>- Which one do you prefer least? (why?)<br>- Is there another word you would prefer?	parents
